# Stereological estimations and neurochemical characterization of neurons expressing GABAA and GABAB receptors in the rat pedunculopontine and laterodorsal tegmental nuclei

**DOI:** 10.1007/s00429-021-02375-9

**Published:** 2021-09-12

**Authors:** Esther Luquin, Beatriz Paternain, Inés Zugasti, Carmen Santomá, Elisa Mengual

**Affiliations:** 1 Department of Pathology, Anatomy, and Physiology, School of Medicine, University of Navarra, Ed. Los Castaños, Irunlarrea 1, 31008 Pamplona, Spain; 2 IdiSNA, Navarra Institute for Health Research, 31008 Pamplona, Spain

**Keywords:** Basal ganglia, GAD67, Vglut2, In situ hybridization, Gait, Reward

## Abstract

To better understand GABAergic transmission at two targets of basal ganglia downstream projections, the pedunculopontine (PPN) and laterodorsal (LDT) tegmental nuclei, the anatomical localization of GABAA and GABAB receptors was investigated in both nuclei. Specifically, the total number of neurons expressing the GABAA receptor γ2 subunit (GABAAR γ2) and the GABAB receptor R2 subunit (GABAB R2) in PPN and LDT was estimated using stereological methods, and the neurochemical phenotype of cells expressing each subunit was also determined. The mean number of non-cholinergic cells expressing GABAAR γ2 was 9850 ± 1856 in the PPN and 8285 ± 962 in the LDT, whereas those expressing GABAB R2 were 7310 ± 1970 and 9170 ± 1900 in the PPN and LDT, respectively. In addition, all cholinergic neurons in both nuclei co-expressed GABAAR γ2 and 95–98% of them co-expressed GABAB R2. Triple labeling using in situ hybridization revealed that 77% of GAD67 mRNA-positive cells in the PPT and 49% in the LDT expressed GABAAR γ2, while 90% (PPN) and 65% (LDT) of Vglut2 mRNA-positive cells also expressed GABAAR γ2. In contrast, a similar proportion (~2/3) of glutamatergic and GABAergic cells co-expressed GABAB R2 in both nuclei. The heterogeneous distribution of GABAAR and GABABR among non-cholinergic cells in PPN and LDT may give rise to physiological differences within each neurochemical subpopulation. In addition, the dissimilar proportion of GABAAR γ2-expressing glutamatergic and GABAergic neurons in the PPN and LDT may contribute to some of the functional differences found between the two nuclei.

## Introduction

The pedunculopontine (PPN) and the laterodorsal (LDT) tegmental nuclei are two complex brainstem structures characteristically containing cholinergic cells (Mesulam et al. 1983), which form an anatomical continuum in the mesopontine tegmentum and are considered as a functional unit working in an integrated fashion (Mena-Segovia 2016). Some of their functions, like locomotion and motor adaptive control, action selection and reward, are related to their reciprocal connections with basal ganglia structures (Mena-Segovia et al. 2004; Roseberry et al. 2016; Takakusaki et al. 2016; Gut and Winn 2016). Specifically, both the PPN and LDT receive abundant projections from the output nuclei of the basal ganglia together with minor projections from the striatum and lateral globus pallidus (Cornwall et al. 1990; Edley and Graybiel 1983; Rye et al. 1987; Shink et al. 1997; Steininger et al. 1992), all of which are GABAergic. However, despite the fact that GABA actions on the PPN have long been known from electrophysiological and pharmacological studies (Ikeda et al. 2004; Nandi et al 2002; Saitoh et al 2003; Torterolo et al 2002), specific studies about the anatomical distribution of GABA receptors (GABARs) in the PPN or LDT have not been carried out so far.

In addition, intermingled with their characteristic cholinergic neurons, PPN and LDT also comprise abundant glutamatergic and GABAergic cells (Wang and Morales 2009; Mena-Segovia et al. 2009; Luquin et al. 2018). Specifically, some of these non-cholinergic cells seem to be the main target of GABAergic projections from the basal ganglia output nuclei (Grofova and Zhou 1998; Mongia et al. 2015; Sherman et al. 2015; Roseberry et al. 2016; Caggiano et al. 2018). However, little is known about the relative distribution of nigral or pallidal terminations on each of the three neurochemical subpopulations of the PPN and LDT, or about the anatomical localization of postsynaptic GABARs potentially mediating the direct inhibitory control from these projections. As a first step to better understand the interactions between the downstream projections of basal ganglia output nuclei and the PPN and LDT at the cellular level and the neural circuitry within these nuclei, we aimed to anatomically confirm the presence of GABA receptors within the PPN and LDT, and to quantitatively determine their relative distribution among the three cell phenotypes in the two nuclei.

Electrophysiological and pharmacological studies have demonstrated the presence of functional GABAA and GABAB receptors in PPN and LDT (Saitoh et al. 2003; Ikeda et al. 2004; Ulloor et al. 2004; Pal and Mallick 2004; Datta 2007; Heinmiller et al. 2009; Ye and Garcia-Rill 2009; Kohlmeier and Kristiensen 2010; Fogel et al. 2010; Kohlmeier et al, 2013). GABAA receptors (GABAARs) are ligand-gated Cl ion channels structurally assembled from five individual protein subunits which include α1-6, β1-3, γ1-3, δ, ε, π, and τ (Olsen and Sieghart 2009). The most common combination of subunits in GABAARs, however, is triplet α1/β2/γ2, which is detected in various cell types in the CNS (Fritschy et al. 1992; McKernan and Whiting 1996). GABAB receptors (GABABRs) are heterodimers formed by the co-assembly of two subunits, GABAB1 and GABAB2, that couple to G-proteins through second messenger pathways and act as modulators of GABA transmission (Bowery et al. 2002). Whole-brain studies on the distribution of either GABAAR or GABABR subunits reported the presence of specific subunits in the rat PPN or LDT in relation to cell bodies, neural processes or neuropil (Fritschy and Möhler 1995; Margeta-Mitrovic et al. 1999; Pirker et al. 2000). However, a specific analysis of GABAAR or GABABR distribution in the PPN and LDT has not been carried out thus far.

Finally, although the PPN and LDT share many common input structures and efferent target areas, both afferent and efferent projections are often topographically organized at the regional level (Dautan et al. 2016a; Hallanger et al. 1987; Oakman et al. 1995; Woolf and Butcher 1986; Xiao et al. 2016) and also at the cellular one (Dautan et al. 2016b), supporting their specific participation in separate circuits as well as their partially different roles (Mena-Segovia 2016). Thus, the aims of the present study were: to assess the expression of GABAARs and GABABRs in each cell phenotype of both nuclei, to estimate with unbiased stereological methods the mean number of GABAAR- and GABABR-expressing cells in the three cell subpopulations, and to investigate whether differences in these estimations were present between the PPN and LDT.

Previous studies have shown that the vast majority of GABAAR in brain contain at least one α subunit variant, along with the β2,3- and γ2-subunits (Pirker et al. 2000;Fritschy and Möhler 1995) and, in general, α1 and γ2 are two of the predominating subunits in the brainstem (Fritschy and Möhler 1995). Thus, for the anatomical detection of GABAAR in the PPN and LDT we used immunoreactivity against α1 and γ2 GABAAR subunits, whereas immunoreactivity against the GABAB R2 subunit was used for the GABABR. Subsequently, the total numbers of cells expressing the GABAAR γ2- and GABAB R2 subunits in both nuclei were estimated using stereological methods and the neurochemical phenotypes of GABAAR γ2- and GABABR R2-expressing cells were investigated. Our results demonstrate the abundant expression of both GABAAR and GABABR in the three cell subpopulations of the PPN and LDT. At the same time, they reveal a heterogeneous distribution of both GABAAR and GABABR among glutamatergic and GABAergic cells that suggests the existence of functionally different subsets within those two subpopulations. Finally, they show marked differences between PPN and LDT regarding GABAAR subunit composition in the cholinergic cell population, as well as in relation to the synaptic versus extrasynaptic location of GABAARs in the non-cholinergic subpopulations. Some of these differences in the mechanisms of inhibitory control of specific cell subsets within the two nuclei may contribute to some of the functional differences observed between them.

## Experimental procedures

### Animals and tissue preparation

Adult male Wistar rats (*n* = 22, 250–350 g; Harlan, Barcelona) were used in this study. Only males were used to minimize potential numerical differences between sexes, while also trying to minimize the number of animals used; variations in receptor subunit localization and distribution, however, are unlikely to be present between males and females. All experimental procedures were carried out in accordance with the guidelines of the National and European Council on the use of animals for research (RD 53/2013 and 2010/63/EC). The experimental design was approved by the Ethical Committee for Animal Testing of the University of Navarra.

The rats were deeply anesthetized with a mixture of ketamine (228 mg/kg, Imalgene 50 mg/mL, Merial Laboratories, France), xylazine (31.2 mg/kg; Rompún 2%, Bayer Health Care, Spain), and atropine (0.22 mg/kg, i.p.; Atropina, Braun Medical SA, Barcelona, Spain), and transcardially perfused with 60 mL of heparin saline (1000 U/mL), and followed by 800 mL of 4% paraformaldehyde in 0.1 M phosphate buffer (PB, pH 7.4), and finally with 500 mL of 10% sucrose in PB. The brains were postfixed in 4% paraformaldehyde for 1 h and then transferred to a 30% solution of sucrose in PB until they sank at 4 °C. Finally, all brains were sectioned in the coronal plane using a freezing microtome, and the 40 μm-thick sections serially collected in cryoprotection solution containing 20% glycerin and 2% dimethyl sulphoxide in PB.

Animals specifically used for in situ hybridization (ISH) were perfused with 250 mL of saline Ringer’s solution followed by 800 mL of cold fixative containing 4% paraformaldehyde in 0.1 M phosphate buffer saline (PBS), pH 7.4 The brains were postfixed in the same fixative solution at 4 ºC overnight and immersed in the cryoprotection solution for 24 h, also at 4 °C (Rosene et al. 1986). All solutions were treated with 0.1% of diethylpyrocarbonate (DEPC, Sigma) and autoclaved prior to their use.

One series comprising one out of every six sections containing PPN and/or LDT (approximately 9–10 sections per case) was processed in each set of experiments.

## Antibody characterization

Mouse monoclonal antibodies against GABAAR α1 and GABAB R2 and a rabbit polyclonal antibody against GABAAR γ2 were used (Table 1), all of which had been characterized previously. Mouse monoclonal antibodies were developed by UC Davis/NIH NeuroMab Facility in mouse generating hybridomas. The anti-GABAAR α1 mouse monoclonal antibody (clone N95/35 monoclonal IgG2a) recognizes protein amino acids 355–394 (cytoplasmic loop) of the α1 subunit and has been used in immunofluorescence labeling in both rat and mice (Micheva et al. 2010; Eyre et al. 2012). The anti-GABAB R2 mouse monoclonal antibody used here (clone N81/2) recognizes protein amino acids 862–913 of the R2 subunit, and has also been used in immunoblotting and immunofluorescence labeling (i.e., Nassirpour et al. 2010; Broussard et al. 2011; Maity et al. 2012). Finally, the anti-GABAAR γ2 rabbit polyclonal antibody recognizes 30 amino acids of an epitope of the extracellular domain of N-terminus of the rat γ2 subunit. This antibody has been used previously in immunofluorescence and immunohistochemistry studies (Sergeeva et al. 2002; Vassias et al. 2005).

**Table 1:** Primary antibodies used for immunohistochemistry and immunofluorescence

Ab against	Raised in	Dilution	Source	Catalog #
GABAA α1	Mouse (monoclonal)	1:500 (IHC)	NeuroMab	75–136
		1:250 (IF)		
GABAA γ2	Rabbit (polyclonal)	1:2000 (IHC)	Alpha Diagnostic	GAG21-S
		1:500 (IF)		
GABAB R2	Mouse (monoclonal)	1:100 (IHC)	NeuroMab	75–124
		1:500 (IF)		
ChAT	Goat (polyclonal)	1:150 (IF)	Merck Millipore	AB144P
NeuN	Rabbit (polyclonal)	1:1000 (IF)	Merck Millipore	ABN78
NeuN	Mouse (monoclonal)	1:1000 (IF)	Merck Millipore	MAB377

## Dual colorimetric labeling for NADPH-diaphorase staining and GABARs immunocytochemistry

One series of sections per receptor subunit—GABAAR α1, GABAAR γ2, and GABAB R2-, was processed for dual colorimetric labeling to simultaneously visualize the cholinergic cells and immunoreactivity against each GABAR subunit. The histochemical staining for nicotinamide adenine dinucleotide phosphate-diaphorase (NADPH-d) was carried out first to visualize the cholinergic neurons of the PPN and LDT (Vincent et al. 1983). Free-floating sections were incubated in Tris buffer (0.1 M, pH 8) containing 1 mM β-NADPH (Sigma), 1 mM Nitroblue Tetrazolium (Sigma), and 0.3% Triton X-100 (Tx), at room temperature (RT). The histochemical reaction was visually controlled, taking generally ~5 min. After rinsing with PBS and prior to the immunocytochemical procedure the sections were incubated in darkness in a quenching solution containing 0.3% hydrogen peroxide (H_2_O_2_) in 50% ethanol for 30 min at RT, to remove endogenous peroxidase activity. After rinsing, the sections were preincubated for 1 h in a blocking solution containing either 10% of normal horse serum (NHS) and 1% bovine serum albumin (BSA) for GABAB R2- and GABAAR α1-immunoreactivities (GABAB R2- and GABAAR α1-ir), or 4% BSA for GABAAR γ2-ir, followed by incubation in the primary antibody solution. All solutions were prepared in PBS and incubations carried out at RT unless otherwise specified. In the case of GABAB R2 and GABAAR α1, the mouse antibodies were diluted in a solution containing 1% NHS, 1% BSA, and 0.3% Tx and incubated for 48 h at 4 °C; the rabbit anti-GABAAR γ2 solution was prepared in 1% BSA and 0.3% Tx and incubated overnight. Tables 1 and 2 summarize the information about the primary and secondary antibodies used in the present study. After rinsing the sections were incubated with either biotinylated horse anti-mouse in 1% NHS and 1% BSA for 2 h, or goat anti-rabbit IgG in the same solution without NHS. The blocking solutions prior to primary and secondary antibody incubations were the same in all experiments unless otherwise specified. Next, the sections were incubated for 1 h in the avidin–biotin complex (ABC standard, Vector laboratories, Burlingame, CA, USA) and followed by 2X rinses. After pre-equilibrating in 0.1 M Tris HCl pH 7.6, the sections were finally incubated in a diaminobenzidine tetrahydrochloride (DAB) solution containing 0.022% DAB and 0.003% H_2_O_2_ in Tris HCl for 15 min and stopped with Tris HCl; then they were mounted on slides using 1:3 PB, dehydrated through graded alcohols, defatted in xylene, and coverslipped with DPX (VWR, Leuven, Belgium).

**Table 2:** Secondary antibodies used for immunohistochemistry, immunofluorescence or fluorescent in situ hybridization

Label	Against	Raised in	Dilution	Source	Catalog #
Alexa 488	Goat	Donkey	1:250	Molecular Probes	A11055
Alexa 555	Rabbit	Donkey	1:250	Molecular Probes	A31572
Alexa 568	Goat	Donkey	1:250	Molecular Probes	A11057
Alexa 647	Rabbit	Donkey	1:250	Molecular Probes	A31573
Biotinylated IgG	Mouse	Horse	1:250	Vector Laboratories	BA2000
Biotinylated IgG	Rabbit	Goat	1:250	Vector Laboratories	BA1000
Streptavidin Alexa 633			1:500 (IF)	Molecular Probes	S21375
			1:500 (ISH)		

## Dual immunofluorescence labeling

A dual fluorescence labeling protocol was used to investigate the potential colocalization of GABAAR- or GABABR-immunoreactivity (GABAAR- or GABABR-ir) with cholinergic neurons using an immunohistochemistry against choline acetyltransferase (ChAT). To enhance the visualization of GABAAR α1- and GABAB R2-ir, a water-bath heating was carried out for antigen retrieval, and an amplification system was used (TSA® detection kit). Thus, the free-floating sections were rinsed and transferred to a PB solution preheated and maintained at 80ºC for 30 min by a surrounding water-bath. Once the sections were lukewarm they were incubated in the quenching solution mentioned previously, for 20 min (Luquin et al. 2010). After rinsing, the sections were preincubated in their respective blocking solutions, followed by incubation in the primary antibody solution containing either mouse anti-GABAB R2 or mouse anti-GABAAR α1 and goat α-ChAT, which was added 16 h before the end of the incubation. The sections were then incubated in a solution with Alexa Fluor 488 donkey anti-goat IgG, for 2 h in darkness. After rinsing, sections were incubated with biotinylated horse anti-mouse IgG in 1% NHS in TNB [0.5% blocking reagent in TN buffer (0.1 M Tris–HCl, pH 7.5, 0.15 M NaCl)] for 45 min, then rinsed once in TNT buffer (TN, 0.05% Tween 20) and 2X in TN, they were incubated in a solution containing streptavidin-conjugated horseradish peroxidase (1:500, TSA^®^ detection kit) in TNB buffer for 30 min, followed by incubation for 10 min with Biotin–tyramide (1:250 in amplification diluent from TSA™). Finally, the sections were incubated with streptavidin-Alexa 633 for 90 min in TNB, and they were mounted on gelatine-coated slides, air dried, defatted in toluene, and covered with DPX. The sections were then examined under a confocal microscope (LSM 880, Zeiss).

For GABAAR γ2-immunofluorescence detection, neither antigen retrieval nor amplification was needed. Instead, the sections were incubated in the corresponding blocking solution, followed by incubation in the primary antibody solution containing rabbit anti-GABAAR γ2 and goat anti-ChAT, and finally incubated in Alexa Fluor 488 donkey anti-goat IgG to label the cholinergic neurons and Alexa Fluor 555 donkey anti-rabbit IgG to visualize GABAAR γ2 neurons.

## Triple immunofluorescence labeling

After inactivation of the endogenous peroxidase activity and incubation in their respective blocking solutions, the sections were incubated in the primary antibody solution containing either mouse α-GABAB R2 or mouse α-GABAAR α1, while goat α-ChAT and rabbit α-Neuronal nuclear protein (NeuN) were added 16 h before the end of the incubation. To enhance the visualization of GABAAR α1 and GABAB R2, the TSA^®^ Plus fluorescein detection kit was used. Thus, the sections were incubated in a solution with biotinylated horse anti-mouse IgG in 1% NHS in TNB for 45 min and after rinsing in TNT buffer and TN, the sections were incubated in a solution containing streptavidin-conjugated horseradish peroxidase (1:200, TSA^®^ Plus fluorescein detection kit) in TNB for 30 min and then incubated for 10 min with FITC-tyramide (1:100 in amplification diluent from TSA Plus kit). After rinses with PBS, the sections were incubated in the secondary antibody solution containing Alexa Fluor 546 donkey anti-goat IgG and an Alexa Fluor 647 donkey anti-rabbit IgG, and finally mounted.

For GABAAR γ2-immunofluorescence detection, the sections were incubated first in the respective blocking solution and then in a solution containing mouse anti-NeuN, rabbit anti-GABAAR γ2 and goat anti-ChAT. To visualize NeuN, the sections were incubated with a biotinylated horse anti-mouse IgG antibody with 1% BSA for 2 h and then in a streptavidin-Alexa 633 solution for 90 min; subsequently they were incubated in a solution containing Alexa Fluor 488 donkey anti-goat IgG and Alexa Fluor 555 donkey anti-rabbit IgG to visualize the cholinergic neurons and GABAAR γ2-positive neurons, respectively.

## Riboprobe preparation for in situ hybridization techniques

Sense and antisense riboprobes of rat GAD67 and Vglut2 were transcribed as described previously (Erlander et al. 1991; Stornetta et al. 2002a,b; Tillakaratne et al. 1992). GAD67 plasmids were generously donated by Drs. A.J. Tobin and N.J.K. Tillakaratne (Department of Biology, University of California, Los Angeles, CA), while the Vglut2 plasmid was kindly gifted by Drs. R.L. Stornetta and P. Guyenet (Department of Pharmacology, University of Virginia, Charlottesville, VA). The riboprobe synthesis was carried out as previously described (Luquin et al. 2018).

## Triple fluorescence labeling for in situ hybridization and dual immunocytochemistry against GABAB R2 and ChAT

A triple fluorescence labeling protocol was used to investigate the potential colocalization of GABAB R2 with either GAD67 or Vglut2 mRNA, carrying out ISH first followed by immunohistochemistry against ChAT. In these sets of experiments, brain tissue from rats perfused with DEPC-treated solutions was used.

For ISH, we first determined the optimal concentrations of GAD67 and Vglut2 sense and antisense digoxigenin riboprobes, which were 110 and 83 ng/mL, respectively. Then, the selected sections were processed for the triple fluorescent protocol. The ISH was carried out first, following a previously described protocol (Luquin et al. 2018). After rinsing with PBS the sections were processed for immunofluorescence, undergoing first preincubations in the respective blocking solutions for 40 min, followed by an overnight incubation in darkness in a solution containing anti-GABAB R2 and anti-ChAT, in which Tx was specifically omitted. Finally, sections were processed according to the dual immunofluorescence labeling protocol.

## Triple fluorescence labeling for in situ hybridization and dual immunocytochemistry against GABAAR γ2 and ChAT

A similar triple fluorescence labeling protocol was used to investigate the colocalization of GABAAR γ2 with GAD67 and Vglut2 mRNA. The optimal concentrations of GAD67 and Vglut2 sense and antisense biotin riboprobes in these cases were 50 and 194 ng/mL, respectively. Selected sections were then processed for ISH as previously described (Luquin et al. 2018) and the biotin riboprobes incubated with streptavidin-Alexa 633 in TNB in darkness. After this, the sections were processed for immunofluorescence undergoing first a preincubation in the respective blocking solution and followed by an overnight incubation in a solution containing anti-ChAT and anti-GABAAR γ2 antibodies. After that, the sections were incubated in a solution containing 1% BSA, an Alexa Fluor 488 donkey anti-goat IgG and an Alexa Fluor 555 donkey anti-rabbit IgG in PBS, and finally mounted.

## Quantification at the confocal microscope

To analyze the potential colocalization of markers, the full rostrocaudal extent of the PPN or LDT was studied in series of one out of six sections (9–10 sections per experiment). In every section, the whole extent of both PPN and LDT was scanned and photographed with a confocal microscope (LSM 800; Zeiss) using a 40× oil-immersion lens with differential interference contrast. The most peripheral cholinergic neurons were used as reference to establish the nuclear boundaries. A complete series of optical sections (Z-stack) was acquired from every field and the interval between every two slices was adjusted at 0.48 μm. Every Z-stack was then analyzed slice-by-slice to determine the potential colocalization of markers on the same plane; thus, all single and dually labeled cells were counted using Zen lite 2012 software (https://www.zeiss.com/microscopy/int/products/microscope-software/zen-lite.html).

To investigate potential regional differences in the expression of receptors between the anterior and posterior portions of the PPN and LDT, in each animal we calculated the percentage of GAD67- and VGlut2-positive cells co-expressing each GABAR subunit in the rostral sections of each nucleus, and statistically compared it with the percentage calculated in the caudal ones (Wilcoxon signed-rank test). When a series of sections had an uneven number of them, the extra section was systematically assigned to the anterior portion. The same calculations and analyses were carried out for ChAT-positive cells expressing the GABAAR α1 subunit.

## Stereological cell quantification

The optical fractionator method (West and Gundersen 1990; Boyce et al. 2010) was used to obtain the total counts of the different cell subpopulations of PPN and LDT because it allows to establish cell numbers independently of volume estimates, eliminating most potential biases due to tissue shrinkage.

The first section for the neuronal counts was randomly selected from the first six containing the PPN, and then one every other 6 (240 µm) was systematically selected throughout the full rostrocaudal extent of PPN and LDT, resulting on average, in 9 sections per animal. Once processed, the sections were analyzed using an Olympus Bx-UCB microscope (Olympus Optical Co, Europe GmbH, Hamburg, Germany) connected to a digital camera (DP71, Olympus) and supplied with a motorized microscope stage ProScan (Prior Scientific Inc, Rockland, MA). The microscope was guided by a computer supplied with NewCast software (NewCast, v.2.16.1.0; Visiopharm, Denmark) which provided a systematic, random, and uniform sampling of optical disectors across tissue sections. These were first examined at 2×; the closed contours of PPN and LDT were outlined at 10X, and cell counts were obtained using a 100× 1.4 NA oil-immersion objective.

The total cell counts of each neurochemical subpopulation were estimated using the following equation:

$$N\, = \,ssf\, \times \,asf\, \times \,hsf\, \times \,\sum Q^{ - },$$

where *ssf* is the section sampling fraction, *asf* the area sampling fraction, *hsf* the height sampling fraction, and *ΣQ*^*−*^ the cells counted in every region. *ssf* was calculated as *T*/BA, where *T* represents the distance between sections (240 µm), and BA is the block advance or thickness set at the microtome (40 µm; *asf* is calculated as *(Dx *×* Dy)/a,* where *Dx* and *Dy* represent the step length in the *X-* and *Y*-axes (155.57 µm in both cases), and *a* is the counting frame area (6050.7 µm^2^); finally *hsf* is calculated as ^®^*t*_*Q*_^*−*^* /h*, where ^®^*t*_*Q*_^*−*^ is the number-weighted mean section thickness and *h* the height of the disector. Cell number estimations obtained with the optical fractionator design are not affected by tissue shrinkage in the *X-* and *Y*-axes; however, to avoid a potential bias due to differential tissue deformation in the *Z*-axis we used the number-weighted mean section thickness or ^®^*t*_*Q*_^*−*^ (Bermejo et al. 2003; Dorph-Petersen et al. 2001). Finally, as the mean section thickness was 13.6 ± 2 µm, the disector height was set at 9 µm, keeping an upper guard zone of 2 µm and a lower one of variable height (2.5 µm on average). Our counting unit was the equator plane of the cell soma, which is the plane of the cell with most sharp borders and it is normally visible in 1–2 microns thickness at most. A cell was counted if the equator was in focus within the height of the disector, which was automatically signaled by the program, and did not touch the forbidden sides (left and bottom) of the disector frame (Luquin et al. 2018).

The sampling fraction was previously determined in a pilot study so as to ensure that a minimum of 100 cells per case from each neuronal phenotype were counted separately in the PPN and LDT, resulting in a coefficient of error (CE) ≤ 0.1 for each of them. The CE was calculated using Eq. 20 from Gundersen et al. (Gundersen et al. 1999). The number of disectors counted ranged between 4 in the smallest areas, and 46 in the largest ones. The volume (V) of PPN and LDT was calculated following the Cavalieri principle (Gundersen and Jensen 1987) using the formula:

$$V\, = \, \, T\, \times \,a\, \times \,\sum P,$$

where *T* represents the distance between sections (240 µm; *a*, is the area per point (0.024 mm^2^), and *ΣP* is the sum of points counted. Once the volume was calculated, the cell densities *(Nv)* were finally estimated using the formula:

$$Nv\, = \,N/V.$$

## Results

### GABAAR α1 and γ2 subunits and GABAB R2 subunit are expressed in both PPN and LDT

Low to moderate numbers of cells expressing α1-α3 and γ2 GABAAR subunits were found in the PPN (Fritschy and Möhler 1995; Rodríguez-Pallares et al. 2001), which are actually some of the most ubiquitous subunits in the brain. Here, we used the α1 and γ2 subunits (GABAAR α1 and GABAAR γ2, respectively) for GABAAR detection. Regarding GABABR, both R1 and R2 subunits are co-expressed throughout the brain and are required for normal receptor function (Benke et al. 1999; Bettler and Tiao 2006; Charles et al. 2001). Initial trials using antibodies against the two subunits revealed a more robust labeling of the R2 subunit, which was, therefore, selected for GABABR detection.

**Fig. 1:**
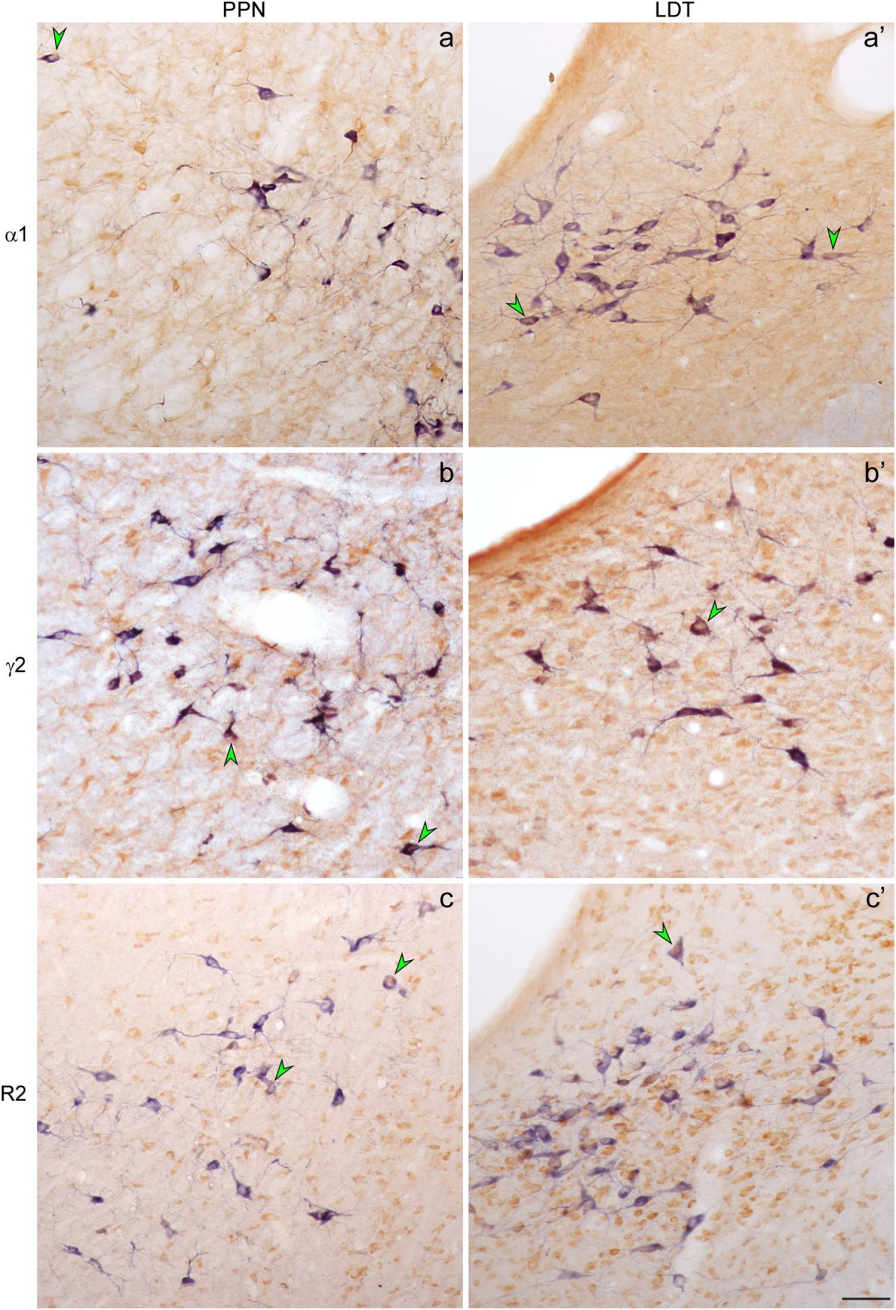
GABAAR α1-, GABAAR γ2-, and GABAB R2-immunoreactive cells in the PPN and LDT. Coronal sections from the PPN (left) and LDT (right) dually labeled for NADPH-d as a marker of cholinergic neurons (blue–black staining), and GABAR subunits (brown reaction product). **a**,** a’** GABAA α1-ir was observed in cell bodies in the PPN and LDT and also in the neuropil. **b**,** b’**, **c**, **c**’ Abundant cells also displayed GABAAR γ2-ir and GABAB R2-ir in both PPN and LDT, immersed within an immunoreactive neuropil. Arrowheads point to neurons with lighter NADPH-d reaction product that also contain brown reaction product, suggestive of dually labeled cells. Scale bar: 100 μm

Coronal brainstem sections were first stained for NADPH-d, a histochemical marker of PPN and LDT cholinergic neurons (Vincent et al. 1983) used to identify the cholinergic subpopulation and delineate PPT and LDT territories, using previously described criteria (Luquin et al. 2017; 2018); secondly, they were immunoreacted against GABAAR α1, GABAAR γ2 and GABAB R2. GABAA α1-immunoreactive cell bodies were observed among NADPH-d stained neurons in both PPN and LDT, immersed within a GABAA α1-positive neuropil (Fig. 1a, a’). Similarly, GABAAR γ2-immunoreactivity (GABAAR γ2-ir, Fig. 1b, b’) and GABAB R2-ir (Fig. 1c, c’) were also observed in a large number of cells and in the surrounding neuropil as well. Thus the expression of the three GABA subunits was confirmed, providing anatomical evidence of the presence of GABAA and GABAB receptors in both PPN and LDT.

### Virtually all GABAAR α1-, GABAAR γ2- and GABAB R2-immunoreactive cells are neurons

**Fig. 2:**
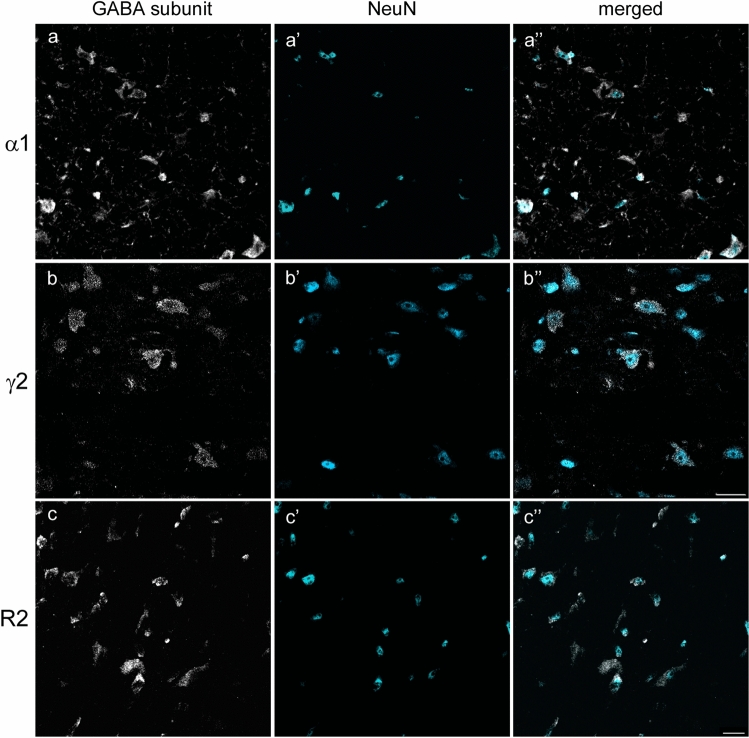
Most GABAAR α1-, GABAAR γ2-, and GABAB R2-immunoreactive cells colocalize NeuN-ir. Confocal images of immunofluorescence labeling in the PPN (**a–a”**, **b–b”**, **c–c’’**) show the high degree of colocalization of either GABAAR α1 (**a’’**), GABAAR γ2 (**b’’**) or GABAB R2 (**c’’**) with NeuN, indicating that the three GABA subunits are almost entirely localized in neurons. All images are the resulting projections of two confocal sections only (0.48 µm each). Scale bars in **b”** and **c”**: 20 µm. Scale bar in **a”**, same as in **c”**

After confirming the expression of the three GABA receptor subunits in the PPN and LDT, we next investigated whether those subunits were localized in neuronal cell bodies. Triple immunolabeling against the neuronal marker NeuN, ChAT, and each GABAR subunit was carried out in separate series from two animals, using the cholinergic neurons for delineation purposes. Confocal images throughout the complete PPN and LDT territories were obtained and all dually labeled cells were counted. The counts revealed that over 90% of GABAAR α1- and GABAB R2-positive cells were NeuN-positive, whereas ~100% of GABAAR γ2-labeled cells co-expressed NeuN-ir in both the PPN and LDT (Fig. 2, Table 3). Given that all three subunits were present in at least 90% of NeuN-positive cells, we have assumed that GABARs are predominantly expressed in neurons and have thus omitted NeuN-ir from subsequent experiments.

**Table 3:** Percentages of co-expression of GABAR subunits with NeuN- and ChAT-ir in PPN and in LDT

	GABAA α1		GABAA γ2		GABAB R2	
	PPN	LDT	PPN	LDT	PPN	LDT
% GABAR^+^/NeuN^+^	95 ± 0.6	90 ± 10	99 ± 0.1	99 ± 0.1	94 ± 2.8	93 ± 0.5
% GABAR^+^/ChAT^+^	46 ± 16*	11 ± 7	100	100	98 ± 1.7	95 ± 5

Data regarding co-expression of GABAR subunits and NeuN were obtained from two animals. Data regarding co-expression of GABAR subunits and ChAT were obtained from five (GABAA γ2 and ChAT) or six animals (GABAA α1 and GABAB R2 and ChAT), depending on the dispersion of the data

*Statistically significant difference between the PPN and LDT (Wilcoxon signed-rank test, *p* = 0.028, *n* = 6)

### PPN and LDT cholinergic neurons co-express GABAA and GABAB receptors

**Fig. 3:**
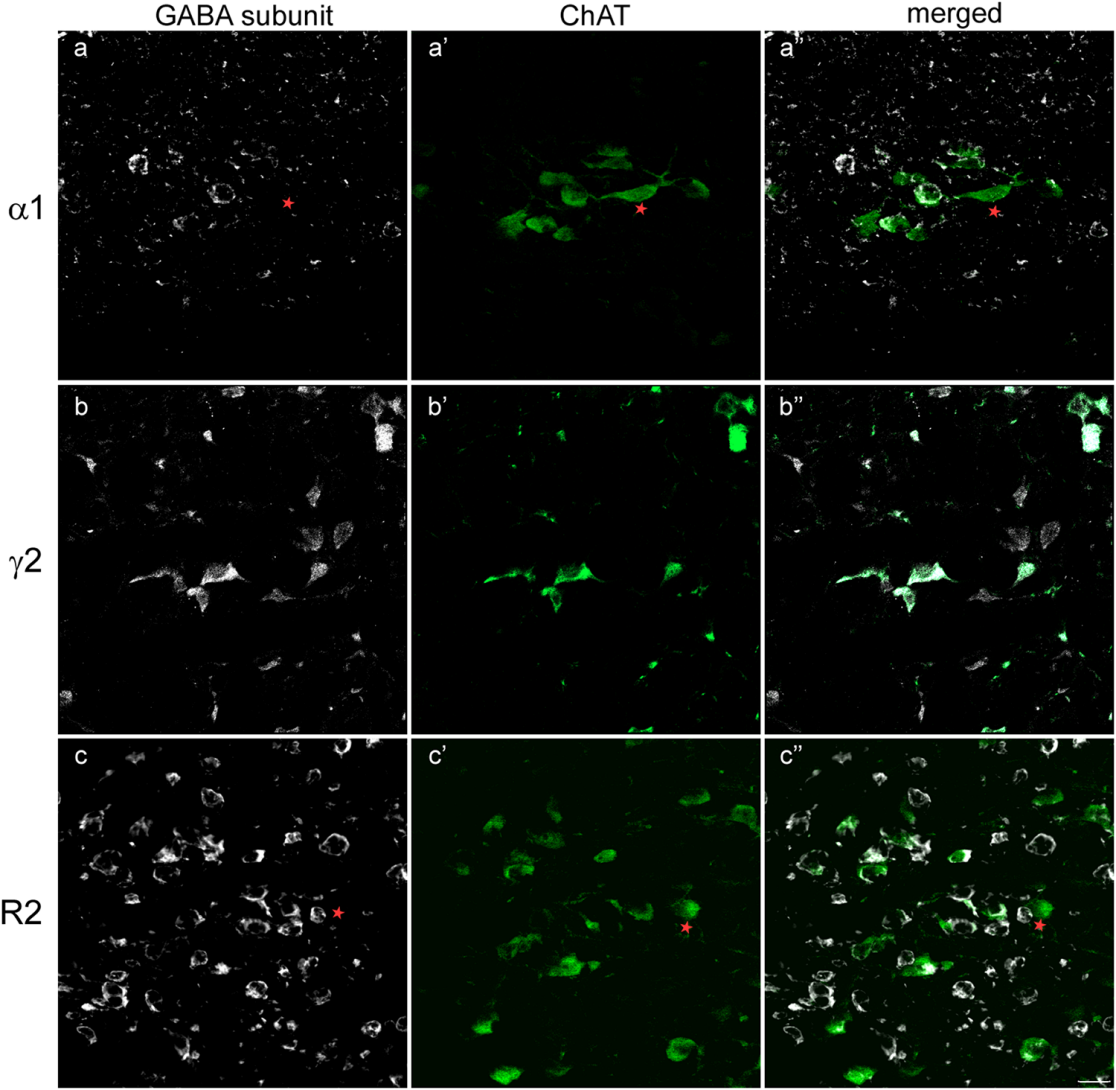
Different degrees of co-expression of ChAT-ir (white) and each of the GABAR subunits (green). **a**–**a’’** Only a subset of ChAT-positive neurons (white) co-expressed the GABAA α1-subunit (green), while others were devoid of GABAA α1-ir (asterisk). **b–b’’** All ChAT-positive neurons co-expressed GABAA γ2-ir, while abundant non-cholinergic GABAA γ2-ir neurons were also visible in the PPN (**a–b’’**). **c**–**c’’** A vast majority of ChAT-positive neurons (white) also expressed GABAB R2-ir (green), while non-cholinergic GABAB R2-positive cells were also observed in the LDT. The asterisk marks a ChAT-positive cell lacking GABAB R2-ir. All images are the resulting projections of two confocal sections only (0.48 µm each). Scale bar: 20 µm

The observation of sections dually labeled against NADPH-d and the GABAR subunits suggested that some NADPH-d-stained neurons were also DAB-containing (Fig. 1). To unambiguously determine whether the cholinergic cells in the mesopontine tegmentum express any of the three GABAR subunits and to what extent, dual immunofluorescence labeling against ChAT and each of the subunits was carried out, and all single and dually labeled cells quantified. Remarkably, the analysis revealed that ~50% of the cholinergic cells in the PPN expressed GABAAR α1 subunit (Fig. 3a–a’), whereas only 11% of them expressed this subunit in the LDT (Table 3), difference that was statistically significant (Wilcoxon signed-rank test, *p* = 0.028, *n* = 6).

In contrast to the α1 subunit, all cholinergic cells in the PPN and LDT expressed the GABAAR γ2 subunit (*n* = 5) (Fig. 3; Table 3b–b’). Finally, GABAB R2 subunit was expressed by > 95% of cholinergic cells in the two nuclei (*n* = 6) (Fig. 3c–c’, Table 3). These results demonstrate that all cholinergic cells in the mesopontine tegmentum express at least one subtype of GABAAR, and virtually all express the GABABR as well.

PPN cholinergic neurons are preferentially located in the caudal portion of the nucleus (Mena-Segovia et al. 2009; Luquin et al. 2018), whereas, in the LDT, these neurons do not show a differential rostrocaudal distribution (Luquin et al. 2018). Given that GABAAR α1 subunit was only present in a subset of the total cholinergic subpopulation in both nuclei, we investigated whether ChAT/GABAAR α1-positive neurons showed a topographical distribution, being differentially present in the rostral versus caudal portions of each nucleus. The comparison between the percentages of dually labeled cells in the rostral versus caudal halves of the two nuclei showed no statistically significant differences either in the PPN (Wilcoxon signed-rank test *p* = 0.753) or in the LDT (Wilcoxon signed-rank test *p* = 0.173).

### Stereological counts of single GABAAR- and GABABR-expressing cells in the PPN and LDT

Next, we estimated the total number of cells expressing either GABAA or GABAB receptors in PPN and LDT using unbiased stereological methods (Fig. 1). γ2 is the most abundant subunit in the brain and forms part of the majority of GABAAR subtypes (Essrich et al. 1998). GABAAR γ2-ir was also more robust and visually more abundant than GABAAR α1 in our dually labeled sections. In light of this we opted for the γ2 subunit as the representative subunit for the GABAA receptor. The delineation of PPN and LDT territories was carried out as reported previously (Luquin et al. 2018). In brief, we outlined the area contained within the most peripherally located NADPH-positive cells in each nucleus. In the PPN no distinction was made between the *pars compacta* and the *dissipata.* Regarding the LDT, both the LDT proper and the ventral LDT or LDTV (Paxinos and Watson 2005) were considered part of a single LDT.

In our dually labeled sections, the brown cells corresponding to single labeled GABAAR γ2- or GABAB R2-positive cells were readily distinguishable from the blue-purple ones stained for NADPH-d (Fig. 1); thus, all single GABAAR γ2- and GABAB R2-positive cells on one hand, and all NADPH-d-stained cells on the other, were counted in the two nuclei (*n* = 5; Table 4). The mean total number of NADPH-d positive neurons—indicative of cholinergic cells—was 3390 ± 774 and 3121 ± 550, in the PPN and LDT, respectively. These estimations were similar to those obtained in a previous study where the cholinergic neurons were identified using ChAT-ir (Luquin et al. 2018). Regarding the GABARs, the mean total number of non-cholinergic cells expressing GABAAR γ2 (9850 ± 1800) was significantly higher than that of cells expressing GABAB R2 in the PPN (7310 ± 1970; Mann–Whitney *U* test, *p* = 0.047 *n* = 5; Table 4). In contrast, the mean total number of GABAB R2-positive cells in the LDT was slightly higher than that of GABAAR γ2 (9170 ± 1900 and 8280 ± 960, respectively), but not statistically significant (Mann–Whitney *U* test, *p* = 0.465; *n* = 5).

**Table 4:** Estimated mean number of NADPH-d stained, GABAAR γ2-, and GABAB R2-immunoreactive cells and cell densities in the PPN and LDT

	PPN		LDT	
	Total cell nº	Density (cells/mm^3^)	Total cell nº	Density (cells/mm^3)^
NADPH-d^+^	3390 ± 774	5430 ± 1000	3121 ± 550	9320 ± 1600
GABAAR γ2^+^	9850 ± 1800*	15170 ± 2300	8280 ± 960	23900 ± 4700
GABAB R2^+^	7310 ± 1970	12110 ± 2640	9170 ± 1900	30700 ± 4780

Data are expressed as mean ± SD

*Statistically significant difference between the total number of GABAAR γ2- and GABAB R2-containing cells in the PPN (Mann–Whitney *U* test, *p* = 0.047; *n* = 5). All other comparisons were not statistically significant

### Co-expression of γ2 and R2 subunits in the GABAergic subpopulation of PPN and LDT

**Fig. 4:**
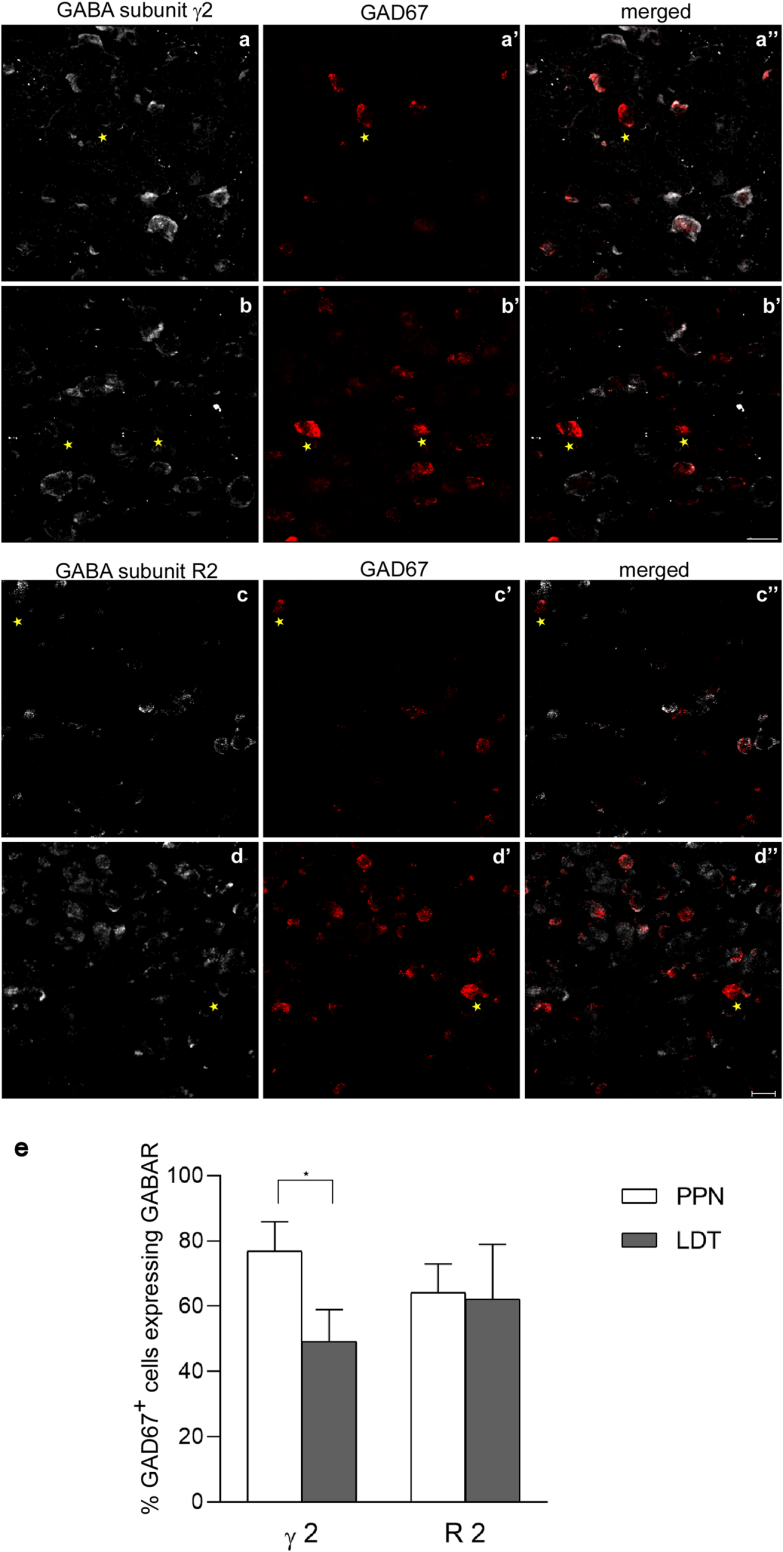
Colocalization of GABAA γ2- and GABAB R2-ir with GAD67 mRNA expression in the PPN and LDT. **a**–**a”** The majority of GAD67-positive neurons (red) in the PPN co-expressed the γ2 subunit (white). An asterisk marks a single GAD67-positive and γ2-negative neuron in the field (**a**–**a”**). **b**–**b”** In the LDT, however, only approximately half of the GAD67-positive neurons (red) co-expressed the γ2 subunit (white). Asterisks mark GAD67-positive/γ2-negative cells. **c**–**c”**, **d–d”** almost two-thirds of GABAergic cells co-expressed the R2 subunit in both the PPN and LDT. Asterisks in **c**–**c”** and **d**–**d”** mark GAD67-positive/R2-negative neurons in the confocal images. All images are the resulting projections of two confocal sections only (0.48 mm each). Scale bars: 20 µm. **e** Graph showing the percentage of colocalization of GAD67 and the two GABA R subunits in the PPN and LDT. The asterisk indicates a statistically significant difference between γ2-expressing GABAergic cells in the PPN and LDT

PPN and LDT contain both glutamatergic and GABAergic cells in addition to the cholinergic ones (Wang and Morales 2009; Luquin et al. 2018). Once we had estimated the mean total number of γ2 and R2-expressing non-cholinergic cells, we investigated the neurochemical phenotype of GABAAR- and GABABR-expressing neurons in both nuclei. To determine the number of GABAergic cells expressing each of the two subunits, ISH against the GABA synthetic enzyme GAD67 was carried out in combination with dual immunofluorescence against either GABAAR γ2 (*n* = 7) or GABAB R2 (n = 5) and ChAT. The semi-quantitative analysis of all dually labeled cells showed a higher percentage of GABAergic cells expressing GABAAR γ2 in the PPN than in LDT (77% ± 8.9 and 49% ± 9.8, respectively; Fig. 4, Table 5), difference that was statistically significant (Wilcoxon signed-rank test, *p* = 0.018; Fig. 4e). In contrast, the percentage of GAD67-positive cells expressing the GABAB R2 subunit was similar in the two nuclei, representing approximately two-thirds of the total GABAergic subpopulation of PPN and LDT (64% ± 8.7 and 62% ± 16.8, respectively; Fig. 4e, Table 5). Together, these results reveal that there are at least two different subpopulations of GABAergic neurons with respect to their GABAAR and GABABR profiles; in addition, they also revealed that the proportion of GABAergic cells containing the γ2 subunit is different in the PPN and LDT.

**Table 5:** Percentages of co-expression of GABAR subunits in the GABAergic and glutamatergic subpopulations of PPN and LDT

	GABAAR γ2		GABAB R2	
	PPN	LDT	PPN	LDT
% GAD67^+^/GABAR^+^	77 ± 8.9*	49 ± 9.8	64 ± 8.7	62 ± 16.8
% Vglut2^+^/GABAR^+^	90 ± 3.7^†^	65 ± 10.1	61 ± 12.5	61 ± 6.4

The number of cases used was *n* = 5 except in the case of the dual expression of GAD67 and GABAAR γ2 in which *n* = 7

*Statistically significant difference between the PPN and LDT (Wilcoxon signed-rank test, *p* = 0.018)

^†^Statistically significant difference between the PPN and LDT (Wilcoxon signed-rank test, *p* = 0.043)

Lastly, we investigated whether regional differences were present in the distribution of GAD67-positive cells expressing either GABAARs or GABABRs. To do so, the percentage of dually labeled cells among GAD67-positive cells in the anterior portion of PPN and LDT was compared to that in the posterior portion. Regarding GAD67/γ2-positive cells, no statistically significant differences were found between the rostral and caudal portions of either PPN (Wilcoxon signed-rank test *p* = 0.735) or LDT (Wilcoxon signed-rank test *p* = 0.398). Similarly, no statistically significant differences were found in the rostrocaudal distribution of GAD67/GABAB R2-positive cells either in the PPN (Wilcoxon signed-rank test *p* = 0.225) or LDT (Wilcoxon signed-rank test *p* = 0.686). These results indicate that, although GABAergic cells are preferentially located in the rostral portion of both PPN and LDT (Mena-Segovia et al. 2009; Luquin et al. 2018), there are no regional rostrocaudal differences in the relative distribution of GABAergic cells co-expressing either GABAAR γ2 or GABAB R2.

### Co-expression of γ2 and R2 subunits in the glutamatergic subpopulation of PPN and LDT

**Fig. 5:**
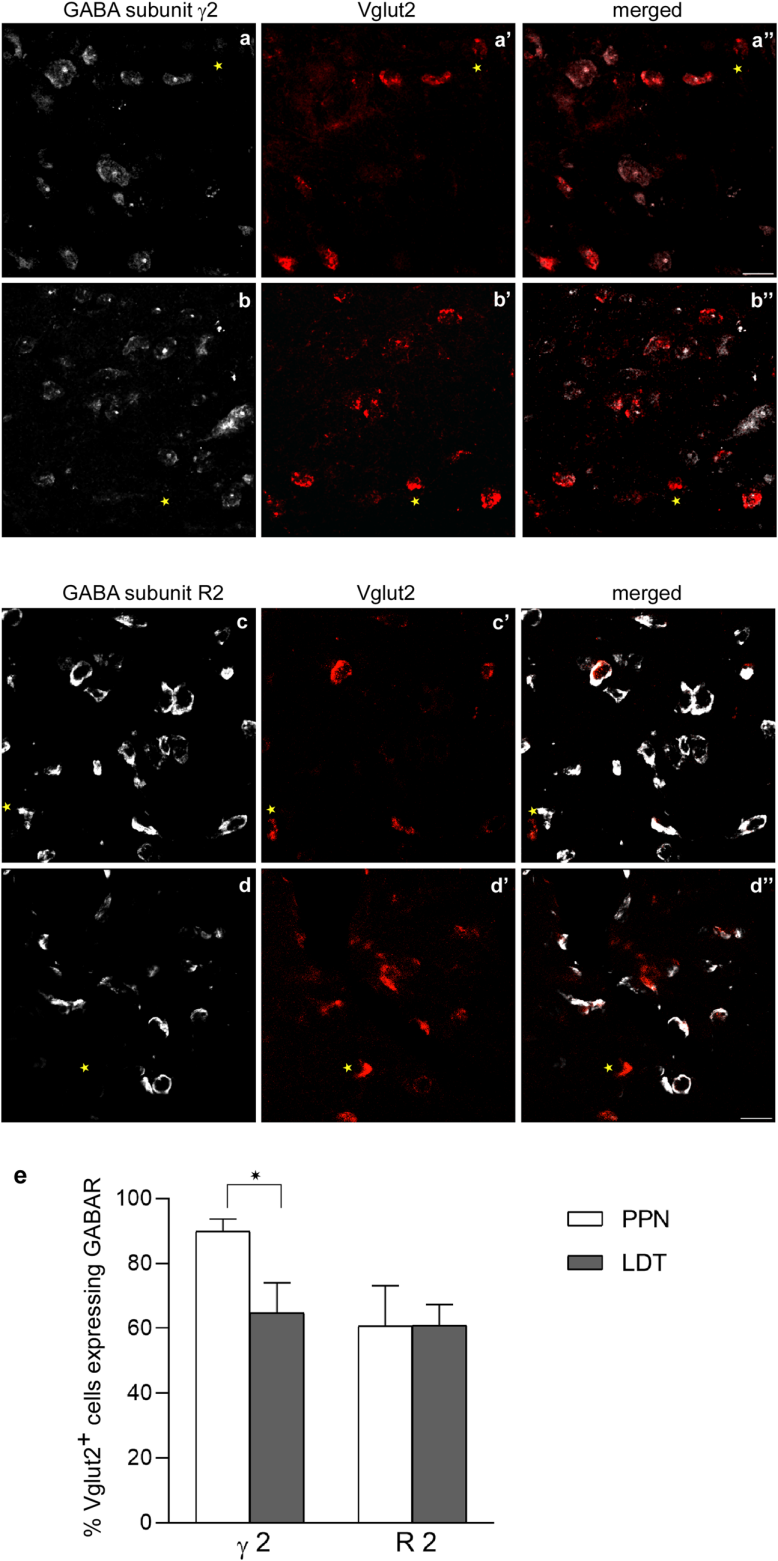
Colocalization of GABA subunit γ2 and GABAB R2 with Vglut2 mRNA expression in the PPN and LDT. **a**–**a”** The vast majority of VGlut2-positive neurons (red) co-expressed the γ2 subunit (white) in the PPN. The asterisk marks a single VGlut2-positive/γ2-negative neuron (**a**–**a”**). **b**–**b”** About two-thirds of VGlut2-positive neurons (red) also co-expressed the γ2 subunit (white) in the LDT. An asterisk marks a Vlgut2-positive neuron clearly lacking the R” subunit. **c**–**c”**, **d**–**d”** about two-thirds of Vglut2-positive neurons co-expressed the R2 subunit both in the PPN and LDT. Asterisks mark Vglut2-positive neurons lacking the GABA receptor subunit. All images are the resulting projections of two confocal sections only (0.48 mm each). Scale bar: 20 µm. **e** Graph showing the percentage of colocalization of VGlut2 and the two GABA R subunits in the PPN and LDT. The asterisk indicates a statistically significant difference between γ2-expressing glutamatergic cells in the PPN and LDT

Next we analyzed the glutamatergic subpopulation; in this case, ISH against the VGlut2 was carried out in combination with dual immunofluorescence against either GABAAR γ2 or GABAB R2 and ChAT (*n* = 5) to determine the number of glutamatergic cells expressing each GABAR subunit. The semi-quantitative analysis of all dually labeled cells revealed that 90% ± 3.7 of glutamatergic cells in the PPN expressed GABAAR γ2 whereas only 65% ± 10.1 of those in the LDT did (Table 5). The statistical comparison between the two showed a significantly higher proportion of co-expression in the PPN (Wilcoxon signed-rank test, *p* = 0.043; Fig. 5e, Table 5). Regarding the GABAB R2 subunit, the same proportion of glutamatergic cells expressed this subunit in the PPN and LDT, which represented approximately two-thirds of the total (61% ± 12.5 and 61% ± 6.4, respectively; Fig. 5e, Table 5). Thus, two subpopulations of glutamatergic neurons can be distinguished with regard to their GABAAR γ2 and GABAB R2 contents; also, in line with the observations on the GABAergic cells, the proportion of GABAAR γ2-expressing glutamatergic cells was different in the PPN and LDT. Finally, comparisons were also carried out between the respective percentages of glutamatergic cells expressing GABAARs and GABABRs in the anterior versus posterior portions of the PPN and LDT. Regarding VGlut2/γ2-positive cells, no statistically significant differences were found between the rostral and caudal portions of either the PPN (Wilcoxon signed-rank test *p* = 0.080) or LDT (Wilcoxon signed-rank test *p* = 0.138). Similarly, no statistically significant differences were found in the rostrocaudal distribution of VGlut2/GABAB R2-positive cells either in the PPN (Wilcoxon signed-rank test *p* = 0.345) or LDT (Wilcoxon signed-rank test *p* = 0.500).

These results indicate that, although glutamatergic cells are preferentially located in the caudal portion of the PPN (Luquin et al. 2018), there are no regional rostrocaudal differences in the relative distribution of glutamatergic cells co-expressing either GABAAR γ2 or GABAB R2 in this nucleus, and the same stands for the LDT.

### Relative expression of γ2- and R2 subunits in PPN and LDT

**Fig. 6:**
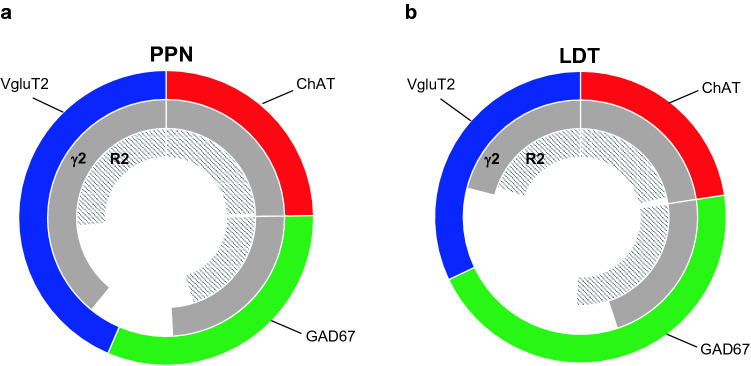
Estimated percentage of the GABAAR γ2 and GABAB R2 subunits in the total PPN and LDT, with respect to the relative percentages of the three cell phenotypes in each nucleus. The outermost circular crown represents the percentual estimations of glutamatergic (blue), cholinergic (red) and GABAergic (green) subpopulations in the PPN (**a**) and LDT (**b**), as previously estimated (Luquin et al. 2018); the middle one (solid gray) represents the estimated percentage of co-expression of GABAAR γ2 in the three subpopulations in the PPN (**a**) and LDT (**b**), whereas the innermost circular crown (gray-lined) represents the estimated percentage of co-expression of GABAB R2 in the same subpopulations of the two nuclei

Previous studies from our laboratory (Luquin et al. 2018) estimated the relative composition of PPN and LDT with respect to their three different neurochemical cell subpopulations (Fig. 6, outermost circular crown). This result, together with the present data regarding the extent of co-expression of GABAAR γ2 subunit and GABAB R2 subunit in each of the three cell phenotypes enabled us to infer the relative presence of each subunit in the global cell population of both PPN and LDT. Overall, GABAAR γ2 subunit (Fig. 6, gray middle circular crown) is present in 87% of PPN cells but only in 65% of LDT cells. This difference is due on one hand, to the fact that the percentages of both glutamatergic and GABAergic cells co-expressing the GABAAR γ2 subunit in the PPN were higher than those in the LDT (Table 5). In addition, the percentage of co-expression of the γ2 subunit in the glutamatergic cells was higher than in GABAergic cells in both nuclei (Table 5), and the former subpopulation is larger in the PPN than in the LDT, as previously reported (Luquin et al. 2018).

In contrast, the percentage of GABAB R2-expressing cells in the PPN and LDT (shaded innermost circular crown) was very similar (70 and 68%, respectively), as the percentages of co-expression of the R2 subunit in each phenotype were virtually the same in the two nuclei.

## Discussion

Here we have provided the first anatomical evidence of the presence of GABAAR and GABAB receptors in the different cell phenotypes in the PPN and LDT. A detailed quantitative analysis of GABAAR γ2 and GABAB R2 subunits in the two nuclei was carried out, and the mean total number of cells expressing each subunit was estimated. Furthermore, we also quantified the number of cells expressing each receptor subunit within each neurochemical subpopulation. While all cholinergic cells in both nuclei expressed GABAAR γ2 and GABAB R2, only a certain proportion of the total glutamatergic and GABAergic cells expressed the γ2 subunit, suggesting that two functionally heterogeneous subsets of cells may coexist among cells of the same phenotype. The proportion of GABAAR γ2-expressing cells in the two non-cholinergic cell subpopulations differed between the PPN and LDT. This, together with a marked difference between the two nuclei regarding the presence of GABAAR α1 subunit in the cholinergic cells might contribute to functional differences between the PPN and LDT.

### Methodological considerations

All antibodies used had been characterized previously (see “Experimental procedures”). Immunohistochemical detection is known to critically depend on epitope accessibility (Lorincz and Nusser 2008). In our colorimetric sections epitope accessibility was confirmed, as all α1-, γ2- and R2-immunolabelings were observed at all levels of the z-stacks, ensuring that the antibody had penetrated completely throughout the sections. However, for fluorescence immunolabeling of α1 and R2 subunits, a pretreatment of water-bath heating was carried out for antigen retrieval, to enhance the labeling (see “Experimental procedures”). Immunoreactivity against α1 was still light, but a low affinity of the α1 antibody was disregarded, as intense α1-ir was observed in the median raphe nucleus and ventral tegmental area in the same sections as previously reported (Fritschy and Möhler 1995; Pirker et al. 2000), confirming the specificity and sensitivity of the antibody used here. Such low immunoreactivity against α1 may reflect the low presence of this subunit in cell bodies of the PPN and LDT, as reported (Fritschy and Möhler 1995), and may well account for its scarce or null detection (Pirker et al. 2000; Rodríguez-Pallares et al. 2001).

### GABAAR γ2 subunit in PPN and LDT

Whole-brain immunocytochemical studies reported the presence of immunoreactive cell bodies expressing diverse GABAAR subunits in the PPN and LDT (Fritschy and Möhler 1995; Pirker et al. 2000), while pharmacological and electrophysiological studies specifically showed the presence of functional postsynaptic GABAA receptors in the two nuclei (Kohlmeier and Kristiensen 2010; Ikeda et al. 2004; Saitoh et al. 2003). The present results confirm the findings of the electrophysiological studies regarding the presence of GABAA receptors in PPN and LDT (Kohlmeier and Kristiensen 2010; Ikeda et al. 2004; Saitoh et al. 2003); moreover, and consistent with Fritschy and Möhler (1995), these results confirm the presence of somatic GABAAR α1 and γ2 subunits in the PPN and LDT and extend the anatomical data estimating that 87% of PPN cells and 65% of LDT cells specifically express the γ2 subunit (Fig. 6).

Regarding the neurochemical phenotype of GABAAR γ2-expressing cells, we have shown that all mesopontine cholinergic cells express GABAAR γ2. The specific presence of functional GABAARs in mesopontine cholinergic cells was reported first in the PPN (Saitoh et al. 2003) and then in the LDT (Kohlmeier and Kristiensen 2010); more recently, indirect anatomical evidence was provided by the beautiful demonstration of GABAAR γ2-ir in cholinergic axon terminals in the pontis oralis nucleus (Liang and Marks 2014), most likely originating from cholinergic PPN and LDT neurons (Mitani et al. 1988; Semba et al. 1990; Shiromani et al. 1988). Consistent with this, we have confirmed the presence of GABAAR in the cholinergic subpopulation of the PPN and LDT, and specifically of the γ2 subunit (Liang and Marks 2014), and have extended these data to the 100% of cells.

GABAAR subtypes can be roughly subdivided into those preferentially located at synaptic sites versus those located extrasynaptically. The ones located synaptically are those containing mainly the α1, α2, and α3 subunits, together with β subunit variants and the γ2 subunit (Fritschy and Panzanelli 2014), being the γ2 subunit essential for synaptic localization and clustering of the receptor subtypes (Essrich et al. 1998). At the same time, synaptically located GABAAR subtypes undergo phasic activation and are responsible for fast inhibition. Our results indicate that all cholinergic neurons are endowed with GABAARs mediating fast inhibition. This does not preclude that additional GABAARs containing γ1, γ3 or δ subunits associated to subtypes located extrasynaptically, might also be present in this cell phenotype. Actually, the presence of the δ subunit in LDT cholinergic cells has been strongly suggested after electrophysiological studies (Kohlmeier and Kristiensen 2010).

Regarding non-cholinergic cells, 10% of glutamatergic neurons in the PPN and 35% in the LDT were γ2-negative, while 23% (PPN) and ~50% (LDT) of the GABAergic ones also lacked γ2. The γ2 subunit is associated with the vast majority of GABAAR subtypes (Fritschy and Möhler 1995) while receptors in which the γ2 is replaced by γ1, γ3 or δ are less abundant. However, single cell bodies expressing the γ3 subunit and moderate numbers expressing the δ subunit have been reported in the PPN (Pirker et al. 2000), suggesting that γ2-negative glutamatergic and GABAergic cell subsets might instead contain either γ3 or δ subunits. Thus far, GABAAR subtypes containing the δ subunit have been predominantly or exclusively found at extrasynaptic sites in cerebellar granule cells and dentate gyrus granule cells (Nusser et al. 1998; Wei et al. 2003). Altogether, the present findings suggest that while a substantial proportion of glutamatergic and GABAergic cells in the PPN and LDT express synaptically located GABAAR subtypes, a proportion ranging from 10 to 50% of the two cell phenotypes does not, likely expressing extrasynaptic GABAARs only. Two subsets of GABAergic cells would thus be present in PPN and LDT: a larger one with cells subject to fast inhibition, and a smaller one, subject to tonic inhibition-mediated control; likewise, a similar segregation would be present among glutamatergic cells as well.

### GABAARγ1 subunit in PPN and LDT cholinergic subpopulations

In addition to the *γ*2 subunit, co-expression of the α1 subunit was also analyzed in the cholinergic subpopulation. Moderate or intense GABAAR α1-ir had been reported in cell bodies of the LDT or PPN, respectively, as well as moderate α2- (PPN) and α3-ir (Fritschy and Möhler 1995). Here, our immunofluorescence analysis revealed that 50% of PPN cholinergic cells and 11% of LDT ones co-expressed the α1 subunit. GABAARs contain two α subunits that can be homologous or heterologous. While the α1α1-containing receptor is by far the most abundant GABAAR subtype in brain (Benke et al. 2004), other combinations, like α1–α3, α1-α2, α3–α3, and α2–α2 coexist in native receptors (Araujo et al. 1996; Benke et al. 2004). Dual immunocytochemical studies have shown that ChAT-positive neurons in the PPN express the α3 subunit, while some scattered cholinergic cells also expressed α2 (Rodríguez-Pallares et al. 2001). Furthermore, cholinergic interneurons in the striatum, septum, diagonal band of Broca and PPN are all α3–positive (Gao et al. 1995; Rodríguez-Pallares et al. 2000, 2001). According to this, it seems plausible that cholinergic neurons express common GABAAR subtypes comprising specific subunits. A detailed study of the expression of GABAAR α1−α3 subunits in mouse cholinergic striatal interneurons has reported that the α3 represented 46% of the total a subunit content, α1, 37% and α2, 17% (Boccalaro et al. 2019). This distribution would be consistent with the reported predominance of α3 in GABAAR in rat cholinergic neurons (Gao et al. 1995; Rodríguez-Pallares et al. 2001), and compatible with a relevant proportion of α1 as well, as reported here, wherein 50% of PPN cholinergic neurons expressed the α1 subunit. Altogether, we may hypothesize that 50% of PPN cholinergic neurons likely contain GABAARs comprising either an α1–α1 combination (generally more frequent, according to Benke et al. 2004) or an α1−α3 one (more likely), whereas the remaining 50% would instead comprise either the α2−α3 combination, or the α3−α3, consistent with the reported data (Fritschy and Möhler 1995; Pirker et al. 2000; Rodríguez-Pallares et al. 2001).

Distinct subunit composition confers specific deactivation and desensitization properties that may profoundly affect synaptic decay kinetics and the capability to sustain high frequency synaptic inputs (Tia et al. 1996). Actually, the presence of α1 subunit confers a decay of only a few milliseconds to α1 subunit-containing GABAARs (Bartos et al. 2001), but of tens of milliseconds to α3 subunit-containing GABAARs (Eyre et al. 2012). Cells that express GABAARs subtypes containing heterologous α subunits—as seemingly do PPN and LDT cholinergic cells, show a variability in decay kinetics that parallels the α1/α3 ratio in a given cell that would lay somewhere between the extremes seen with α1-only- and α3-only-expressing cell types (Eyre et al. 2012). The fact that a variable proportion of PPN and LDT cholinergic neurons contain at least one α1 subunit while the remaining cells do not, suggests that at least two cholinergic subsets would be present in both nuclei, one endowed with a faster synaptic decay than the other. Differences in the electrophysiological properties of PPN/LDT cholinergic cells have been consistently reported (i.e., Boucetta et al. 2014; Mena-Segovia et al. 2008; Sakai 2012; Takakusaki et al. 1997). It is thus plausible to hypothesize that, differences in GABAAR receptor kinetics between the two subsets may somehow contribute to the overall electrophysiological differences found among these cholinergic cells: to what extent and how, remains to be investigated.

### GABAB receptor in the PPN and LDT

Electron microscopic studies have shown that the two subunits of GABABRs are expressed both presynaptically and postsynaptically in most neurons of basal ganglia nuclei in rat and monkey (Boyes and Bolam 2007 and refs therein). Postsynaptically, both subunits were mainly located at extrasynaptic sites, although some of them were found at symmetrical synapses, likely co-localized with GABAARs (Boyes and Bolam 2007). Regarding the PPN and LDT, electrophysiological and pharmacological studies have evidenced the presence of postsynaptic GABABRs in the two nuclei (Heinmiller et al. 2009; Kohlmeier and Kristensen 2010; Kohlmeier et al. 2013; Ulloor et al. 2004), although to our knowledge, protein expression in the LDT has only been reported in a whole-brain analysis of GABABR-immunoreactivity using the R1 subunit (Margeta-Mitrovic et al. 1999). Here, in line with that study, we have confirmed the presence of postsynaptic GABABRs in the PPN and also in the LDT using the R2 subunit, further supporting that the two GABABR subunits are necessary for functional GABABRs (Benke et al. 1999; Bettler and Tiao 2006; Charles et al. 2001). In addition, we have extended those results determining that virtually all cholinergic cells, as well as two-thirds of the glutamatergic and GABAergic cells in both nuclei, express the R2 subunit.

GABAB receptors are negatively coupled to cyclase adenylate via Gi proteins. This accounts for the delay in the onset of the hyperpolarization mediated by these receptors as well as for their slow time course. As reported here, nearly all cholinergic cells express GABABRs receptors, in addition to the expression of synaptic GABAARs in 100% of them; these findings support that virtually all mesopontine cholinergic cells are not only under fast inhibitory control through GABAARs, but also under slow inhibitory control by GABABRs. Furthermore, the fact that virtually all mesopontine cholinergic neurons express both GABAARs and GABABRs, suggests that single cells contain the two receptor types. Interestingly, a recent study has shown a cross-talk between GABAARs and GABABRs in the mammalian central nervous system in which the activation of GABABR suppressed GABAAR responses, and this disinhibition occurred when both receptors were present on the same cell (Shen et al. 2017). While the outcome of either suppressing or enhancing inhibition of GABAARs may depend on the subunit composition of the GABAARs (Shen et al. 2017 and refs. therein), the same-cell localization of GABAARs and GABABRs in cholinergic cells suggested here provides the anatomical substrate for a potential effect of one receptor type upon the other, in addition to the individual function of each receptor type alone.

Regarding the non-cholinergic cells, two subsets of glutamatergic and GABAergic cells seem to also be present in PPN and LDT in relation to GABABRs, a larger one expressing the receptor, and a smaller one lacking the R2 subunit, and thus most likely lacking functional GABABRs. If we combine the expression of GABAARs and GABABRs (Fig. 6), glutamatergic cells in either nucleus might potentially be divided into up to four subsets according to their particular GABA receptor profile: GABAA + /GABAB + , GABAA + /GABAB−, GABAA−/GABAB + or GABAA−/GABAB−. Similarly, up to four subsets of GABAergic cells may potentially exist. The differential GABA receptor profile of each subset most likely confers particular electrophysiological properties to the subset within each cell phenotype. Electrophysiological studies in vivo in the PPN have reported a variety of firing patterns in non-cholinergic cells, corresponding to both presumed excitatory and inhibitory cells (Mena-Segovia et al. 2008; Ros et al. 2010; Sakai 2012); in one of those studies, at least three different functional subsets were identified: tonically firing, irregular, and quiescent neurons which fired in different phases of cortical activity (Ros et al. 2010). In addition, heterogeneous responses were also observed when recording from GABAergic neurons in the same region during spontaneous locomotion, “indicating additional complexity in the composition and function of this subpopulation” (Roseberry et al. 2016). How the diversity in GABA receptor profiles among glutamatergic and GABAergic cells and their particular dynamics might contribute to the electrophysiological differences observed among those cell subpopulations deserves further research.

### Differential expression of GABA receptors in the PPN and LDT

Some of the data reported here revealed relevant differences between the PPN and LDT. One is the substantially lower proportion of α1 subunit in LDT cholinergic cells (11%) compared to PPN (50%), which would imply that nearly 90% of LDT cholinergic cells contain GABAARs comprising either α2–α3 or α3–α3 combinations. The deactivation kinetics of α1β2γ2 recombinant receptors are several fold faster than those of α1β2γ2 ones (Barberis et al. 2007), differences that were replicated in thalamic neurons expressing predominantly the α1 subtype (ventrobasal complex, VB) or the α3 (reticular nucleus, RT) (Mozrzymas et al. 2007 and refs. therein). Furthermore, these differences accounted for the rapid versus slow synaptic currents elicited in the VB and RT cells, respectively; however, the physiological role of these differences between inhibitory postsynaptic currents is not clear (Mozrzymas et al. 2007). These authors suggest that faster inhibitory postsynaptic currents elicited in α1-containing cells make them more appropriate for tasks requiring a high temporal resolution, whereas extremely long lasting inhibitory currents such as those elicited in α3-containing cells, provide a particularly potent mechanism for inhibitory drive (Mozrzymas et al. 2007). How rapid versus slow inhibitory postsynaptic currents elicited in PPN and LDT cholinergic cells, respectively, might affect their overall activity and on a much larger scale, might contribute to their segregated functions (Xiao et al. 2016), is not known. To our knowledge, the activity of the two cholinergic subpopulations has not been directly compared, although the specific activation of PPN versus LDT cholinergic axons resulted in a differential modulation of dopaminergic cells in the ventral tegmental area (Dautan et al. 2016b). Whether the differences reported here contribute to this differential modulation remains to be investigated.

Besides, GABAC receptors, a homopentameric subclass of GABAARs consisting solely of rho subunits (ρ1-3; GABAA-ρR), have been demonstrated on cholinergic LDT neurons in mice (Eliasen et al. 2020; Kohlmeier and Kristiansen 2010) despite their very limited distribution. Assuming that there is little difference between species regarding subunit composition of GABAARs (Gao et al. 1995; Boyes and Bolam 2007), GABAC receptors might be present in the LDT in addition to GABAARs, or ρ subunits might be expressed instead of α1 in association with the γ subunit, as has been shown in some brainstem nuclei both in mice and rat (Frazao et al. 2007; Milligan et al. 2004). Homomeric GABAC receptors, however, would represent an additional mechanism of tonic inhibition in LDT cholinergic neurons, as these receptors have been suggested to be extrasynaptic and activated via spillover of synaptically released GABA (Alakuijala et al. 2006).

Another remarkable difference observed between the PPN and LDT lies in the percentage of non-cholinergic cells containing the γ2 subunit. Thus, while almost 80% of GABAergic cells in the PPN express the γ2 subunit, in the LDT, where the GABAergic subpopulation represents 46% of the total (Luquin et al. 2018), barely 50% of them express this subunit. This means that a majority of GABAergic cells in the PPN are subject to fast postsynaptic currents while only half of them in the LDT are; the other half, likely expressing the δ subunit, would instead be subject to tonic inhibition. The same would be applicable to the glutamatergic cells, as up to one third of them in the LDT lack synaptic GABAARs compared to only 10% of them in the PPN. These differences may relate to the activity and functions of specific glutamatergic and GABAergic cell subsets implicated in distinct subcircuits within the PPN and LDT.

### Neural circuitry in the PPN and LDT in relation to GABA receptors

PPN and LDT receive substantial projections from the output nuclei of the basal ganglia (Cornwall et al. 1990; Hallanger and Wainer 1988; Rye et al. Semba and Fibiger 1992), which in turn send reciprocal connections to these as well as to other components of basal circuits (Bevan and Bolam 1995; Kita and Kita 2011; Dautan et al. 2016a,b). However, the neural circuits within the PPN and LDT remain largely unknown. While the cholinergic and glutamatergic subpopulations seem to have a larger amount of cells directly targeting them from the diverse basal ganglia components (Roseberry et al. 2016; Caggiano et al. 2018; Henrich et al. 2020), the cell-specific targets of nigrotegmental and pallidotegmental projections and their relative distribution among the three cell phenotypes are only partially known. Electrophysiological and pharmacological studies showed that 30% of PPN neurons contacted by nigral fibers were cholinergic, and that all inhibitory postsynaptic potentials evoked by nigrotegmental projections were controlled via GABAARs (Saitoh et al. 2003; Ikeda et al. 2004). In addition, PPN non-cholinergic cells targeted by nigrotegmental projections have been identified as glutamatergic cells (Grofova and Zhou 1998). The data reported here that GABAARs are present in the three cell phenotypes confirms that an inhibitory control may be exerted by nigrotegmental projections on both cholinergic and glutamatergic cells, and to a minor extent, on GABAergic cells (Roseberry et al. 2016). In addition, work from our laboratory has revealed potential synaptic contacts of pallidotegmental fibers arising from both the entopeduncular nucleus and the ventral pallidum onto cholinergic, GABAergic, and glutamatergic cells in the PPN and LDT (Mongia et al. 2015; Mongia 2016); interestingly, the relative proportion of contacts on cholinergic cells was also one-third of them, similarly to the reported electrophysiological data on nigrotegmental projections (Saitoh et al. 2003). Although to our knowledge the receptors implicated in these projections have not been established, we may hypothesize that they will also act via GABAARs receptors.

As far as we know, there is no physiological evidence of what afferent projections may be eliciting GABAB-mediated responses in PPN or LDT in relation to basal ganglia downstream projections. However, there are other GABAergic inputs to the PPN and LDT that could be acting on these receptors, arising from structures that participate in different functional circuits (Roseberry et al. 2016; Caggiano et al. 2018; Henrich et al. 2020). For example, a large number of neurons from the central amygdala and the bed nucleus of the stria terminalis, structures involved in fear-related behavior, heavily innervate both nuclei. Actually a number of excitatory, putative glutamatergic cells in LDT are inhibited via GABABR stimulation originating in local GABAergic interneurons, leading to freezing behavior (Yang et al. 2016). Also, GABAB receptors in the PPN mediate suppression of REM sleep and suppress unitary activity of cholinergic cells which are REM-active in the freely moving rat model (Ulloor et al. 2004). Specifically, GABAergic inputs from the ventrolateral periaqueductal grey controlling PS are projecting to cholinergic and glutamatergic neurons in the ventral LDT (Sapin et al. 2009; Weber et al. 2018). Whether these projections and/or additional local GABAergic neurons (Kroeger et al. 2017) act via GABAB receptors, is something that remains to be determined.

## Conclusions

Downstream projections from the basal ganglia output nuclei directly target the three cell phenotypes in the PPN and LDT, and as we show here, they are all endowed with GABA receptors enabling GABAergic transmission. What specific cell subsets of cholinergic and non-cholinergic cells are actually controlled by these projections, and what local and/or reciprocal connections to basal ganglia components do they establish, is a matter of future research that will contribute to understand the role of PPN and LDT within basal ganglia circuitry.

Marked differences were found between the PPN and LDT in relation to GABAAR subunit composition in the cholinergic subpopulation, as well as to the synaptic versus extrasynaptic GABAARs in their non-cholinergic subpopulations. The significance of GABAAR heterogeneity for brain function is not yet understood. What we know is that the diverse dynamic characteristics of different GABAAR subtypes give rise to distinct types of inhibitory control, some of which may coexist in a given nucleus (Ye et al. 2017), while the presence of GABABRs likely provides additional types of inhibitory control. Additional work on GABAAR subunit combinations and their particular kinetics, as well as on their potential interactions with GABABRs will help understand inhibitory neurotransmission in these nuclei and may lead to a better understanding of functional differences between the PPN and LDT.

## Data Availability

The datasets generated during and/or analyzed during the current study are available from the corresponding author on reasonable request.

## Abbreviations

ABC: Avidin–biotin peroxidase complex
BSA: Bovine serum albumin
ChAT: Choline acetyltransferase
DAB: Diaminobenzidine tetrahydrochloride
DEPC: Diethyl propyl carbonate
GABA: Gamma-aminobutyric acid
GAD: Glutamate acid decarboxylase
HRP: Horseradish peroxidase
ir: Immunoreactive
ISH: Fluorescent in situ hybridization
LDT: Laterodorsal tegmental nucleus
NADPH: Nicotinamide adenine dinucleotide phosphate
NADPH-d: Nicotinamide adenine dinucleotide phosphate-diaphorase
NeuN: Neuronal nuclear protein
PB: Phosphate buffer
PBS: Phosphate buffered saline
PPN: Pedunculopontine tegmental nucleus
RT: Room temperature
scp: Superior cerebellar peduncle
SSC: Standard sodium citrate
TB: Tris-hydrochloric acid buffer
TN: Tris-hydrochloric acid–sodium chloride
TNT: Tris-hydrochloric acid–sodium chloride tween
TSA: Tyramide signal amplification
Tx: Triton X-100
Vglut2: Vesicular glutamate transporter
